# The Optimization of Plasma-Activated Water Treatments to Inactivate *Salmonella* Enteritidis (ATCC 13076) on Shell Eggs

**DOI:** 10.3390/foods8100520

**Published:** 2019-10-21

**Authors:** Chia-Min Lin, Yu-Chi Chu, Chun-Ping Hsiao, Jong-Shinn Wu, Chang-Wei Hsieh, Chih-Yao Hou

**Affiliations:** 1 Department of Seafood Science, National Kaohsiung University of Science and Technology, Kaohsiung 811, Taiwan; cmlin@mail.naku.edu.tw (C.-M.L.); kiki246816@gmail.com (Y.-C.C.); 2 Department of Mechanical Engineering, National Chiao Tung University, Hsinchu 300, Taiwan; pandarugby@nctu.edu.tw (C.-P.H.); chongsin@faculty.nctu.edu.tw (J.-S.W.); 3 Department of Food Science and Biotechnology, National Chung Hsing University, Taichung 402, Taiwan; welson@nchu.edu.tw

**Keywords:** plasma-activated water, *Salmonella*, shell egg

## Abstract

Egg is a regularly consumed food item. Currently, chlorinated water washing is the most common practice used to disinfect eggs, but this process has a negative environmental impact. A new physical technique, plasma-activated water (PAW), has been demonstrated to possess effective antibacterial activities without long-term chemical residue. In this study, air PAW was used to inactivate *Salmonella* enterica serovar Enteritidis on shell eggs. Different combinations of activation parameters, including water sources (reverse osmotic (RO) water, tap water), power (40 W, 50 W, 60 W) and activation time (10 min, 20 min, 30 min), were evaluated. The oxidation–reduction potential (ORP) and pH values of each combination were measured, and their antibacterial activity was tested in a bacterial suspension. Higher antibacterial activities, higher ORP values, and lower pH values were obtained with higher power, longer activation time, and lower water hardness. The antibacterial activities of PAW decreased rapidly by increasing the storage time both at room and refrigeration temperatures. Afterwards, RO water was pre-activated for 20 min at 60 W, and then the eggs inoculated with *S. enteritidis* were placed into PAW for 30 s, 60 s, 90 s, or 120 s with a plasma on-site treatment in the water. More than a 4 log reduction was obtained with 60-s and 120-s treatments. The results showed that the freshness indexes of the eggs treated with PAW were similar to those of the untreated controls and better than those of the eggs treated with commercial processes. In addition, observation under a scanning electron microscope also showed less surface damage of the cuticle on the PAW-treated eggs than on the commercially treated eggs. The results of this study indicate that PAW could be an effective antibacterial agent with less damage to the freshness of shell eggs than commercial methods.

## 1. Introduction

Eggs laid by many different species, including birds, amphibians, and fish, have been consumed by mankind for more than a millennium. Among these types, chicken eggs are by far the most commonly consumed. Typically unfertilized, the chicken egg is a cheap source of nutrients such as amino acids, fatty acids, highly digestible vitamins, and minerals [1,2]. It is well known that foodborne *Salmonella* spp. is the main pathogenic microorganism associated with eggs. Out of 2500 different serovars of *Salmonella enterica*, *S. enterica* serovar Enteritidis (*S. enteritidis*) is the most frequently reported serovar in foodborne illnesses associated with eggs and egg products [3,4,5]. The annual number of foodborne illnesses in the United States was estimated to be 1028 million cases, 19,000 hospitalizations, and 400 deaths. Among the 14 major pathogens, *Salmonella* was one of the top three foodborne pathogens in the United States and other countries around the world, and therefore is a significant public health concern worldwide [6].Thus, inactivating *Salmonella* is a major topic in the egg industry. The current practice for the disinfection and cleaning of eggs is to wash them with 100–200 ppm chlorinated water, alkaline detergent (such as quaternary ammonium salts), hot water, or steam at 5–10 °C above the egg’s surface temperature. Among those processes, chlorinated water is the most commonly used method due to its low cost and high efficacy. However, chlorine is not stable in high temperatures, and easily reacts with organic substances to form a potential carcinogen, trihalomethane [7]. In addition, present-day consumers have a negative perception of chemical treatments. Hence, finding novel techniques to disinfect and clean shell eggs without a potential negative impact and without long-term chemical residue problems is required.

Non-thermal plasma is a new approach to improve microbiological safety without compromising the sensory quality of the treated foods. Its antimicrobial mechanism includes ultraviolet (UV) light, an electromagnetic field, charged particles, and reactive species. Recent studies have demonstrated that non-thermal plasmas can efficiently inactivate a wide range of microorganisms, including fungi, viruses, bacteria, bacterial spores, and biofilms [8,9,10]. Operating a plasma generator inside water triggers the ionization of the water and produces reactive nitrogen species (RNS), reactive oxygen species (ROS), and ozone, which are able to inactivate bacteria [11]. These substances are relatively short-lived and degrade rapidly. Thus, chemical residues in the water are not an issue [12,13]. In addition, the air flowing through the generator to create plasma is also ionized and produces similar substances that are dissolved in the water. The type of water activated by plasma is called plasma-activated water (PAW). PAW has been reported to inactivate foodborne pathogens on the food-contact surface and on food without a negative impact on the environment and human health [14]. Successful examples of inactivating pathogens on food include mushroom [10], strawberry [15], and beef [16]. PAW was also reported to be able to extend the shelf life of shrimp [17]. However, PAW has not been applied to shell eggs. Therefore, the objective of this research was to determine the optimal parameters for generating air PAW considering the variation of water sources, activation power, and time. Subsequently, PAW generated based on the determined parameters was used to inactivate *Salmonella* on egg surfaces, and PAW’s effects to the freshness of treated eggs was also investigated.

## 2. Material and Methods

### 2.1. Bacterial Strain and Cell Suspension Preparation

The most commonly occurring pathogen associated with eggs, *Salmonella enterica* subsp. *enterica* serovar Enteritidis (ATCC 13076), was used. The stock culture was stored in 1:1 glycerol:tryptic soy broth (TSB) at −80 °C. A fresh working culture was prepared by inoculating 200 µL of frozen culture in 10 mL of TSB, which was then incubated at 37 °C for 18–20 h in tryptone soy broth (TSB) twice consecutively. This bacterial strain was maintained at on tryptic soy agar (TSA) and confirmed by a selective media (xylose lysine deoxycholate Agar (XLD)). The bacterial population was maintained around 9 log colony-forming unit (CFU)/mL. All media were purchased from Difco Laboratories (Detroit, MI, USA).

### 2.2. The Preparation of Plasma-Activated Water (PAW)

**Figure 1:**
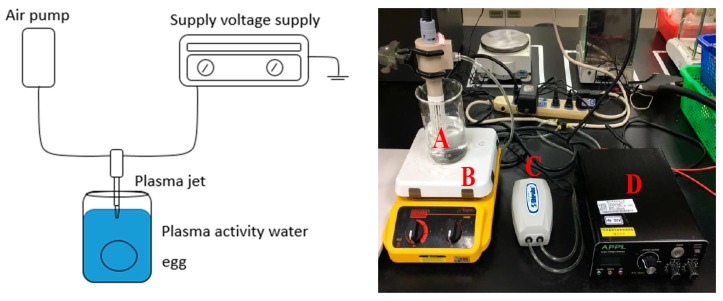
The device for the air plasma-activated water system, including (A) a plasma jet, (B) an electromagnetic stirrer, (C) an air pump, and (D) a high-voltage power supply.

The system to generate non-thermal atmospheric pressure plasma was constructed by the Aerothermal and Plasma Physics Lab (APPL), Department of Mechanical Engineering, National Chiao Tung University (Hsinchu, Taiwan). The major components of the system included a high-voltage power supply, an air pump, and an atmospheric pressure plasma jet (APPJ) (patent, US10,121,638B1) (Figure 1), in which almost all kinds of gases can be applied. The device was specifically designed to activate water by inserting an electrode beneath the water’s surface. The working gas for the plasma was normal air that was pumped by air pump. Based on the preliminary study, the water volume, voltage, frequency, and air flow rate were set at 100 mL, 3.0 kV, 16 kHz, and 5 L/min, respectively. Different combinations of the power consumption (40, 50, 60 Watt), pre-activated time (10, 20, 30 min), and water sources (tap water in Kaohsiung or Pingtung or reserve osmotic water in Kaohsiung and Pingtung) were studied to obtain the optimal combination. After inactivation, the PAW was stored at 4 °C or 25 °C. The oxidation–reduction potential (ORP), pH values, and anti-*S. enteritidis* ability were determined every 24 h for up to 5 days.

### 2.3. Inactivation of Salmonella spp. on Eggs by PAW

Eggs were supplied by a commercial egg farm. All the eggs used in this study were unwashed and laid less than 2 days before testing. Before the study commenced, the natural microorganisms on the egg’s surface were removed via the following procedure, whose effectiveness was verified by preliminary studies: All eggs were washed by RO water, disinfected by 75% ethanol for 20–30 s, rinsed by sterile water, wiped with a sterile paper towel, and finally dried in a laminar hood. The *S. enteritidis* culture was centrifuged at 5000 *g* for 5 min at 4 °C and resuspended in new TSB to achieve a population at 2–3 × 10^9^ CFU/mL; a 100-μL bacterial suspension was placed onto the surface of each egg in 33–35 drops and then air dried for 20–30 min in a laminar hood. The inoculated egg was placed into a beaker containing pre-activated water. The operating plasma was kept active in the water and egg on-site for 30, 60, 90, and 120 s, simultaneously. The water amount was 100 mL. The inoculated eggs placed in sterile water were used as the control. After treatment, the egg sample was transferred into a sterile bag with 100 mL of phosphate buffer saline (PBS, pH 7.2) and then gently rubbed by hand for 2 min. The PBS was decimally diluted serially, and 0.1 mL was spread onto plate count agar (PCA). Triplicate plates were used for each dilution, and the plates were incubated at 37 °C for 18–24 h. Two to three colonies on a PCA plate were randomly re-streaked onto xylose lysine desoxycholate (XLD) agar to confirm that the recovered bacteria were *Salmonella* spp.

### 2.4. Determination of Egg Freshness

After obtaining the optimal procedures to inactive *Salmonella* on the egg’s surface, a fresh batch of eggs was treated using the optimal procedures without inoculation. The eggs were treated with a commercial process that used 100 ppm chlorine as a disinfectant, and the unwashed eggs were used as controls. All eggs were stored at room temperature, and the freshness of the eggs was determined on the testing day (Day 0), as well as 7 and 14 days after treatment. Egg freshness was determined based on the procedures described by Bagheri et al. (2019) [18].

#### 2.4.1. Preparation of Solutions with Different Specific Gravities

Three amounts of NaCl (50, 35, or 15 g) were added into 500 mL of water to produce saline at three gravities (1.078, 1.050, and 1.020 g/mL). The eggs that did not float in the saline with higher gravities were determined to be very fresh. 

#### 2.4.2. Measurements of Yolk and Albumen Indexes

Eggs were cracked and placed on a plate. The height and diameter of the yolk and albumen were measured. Yolk and albumen indexes were defined as the ratio of the height (mm) and diameter (mm). Higher indexes indicated higher degrees of freshness. When the yolk and albumen indexes were below 0.300 and 0.100, the eggs were considered to be non-fresh.

#### 2.4.3. Measurement of Weight-Loss Rate

The weight of the eggs continued to reduce during storage. Thus, highly fresh eggs showed a lower ratio of weight loss. The ratio formula was:

[(the egg weight on Day 0 − the egg weight of the testing day)/the egg weight on Day 0] × 100%(1)

### 2.5. Observations via Scanning Electron Microscope (SEM)

The surface of the unwashed, commercially washed, and PAW (60 W, 20 min, 120 s) treated eggs were used for observation under SEM. The eggs treated with PAW for 120 s were chosen, since this was the longest treatment time and theoretically caused the most damage on the surface. The main focus of SEM observation was the surface cuticle, which is composed of several layers and maintains the freshness of the eggs. Eggshells were collected after treatment, and the inner membranes were removed. After being cleaned by deionized water, the shells were dried at 100 °C for 24 h and then stored in a desiccator. The shells of the inoculated eggs with *S. enteritidis* (as described previously) were collected and observed under SEM to examine the damage of the bacterial cells. After removing the inner membrane, the shells were immersed in a phosphate buffer containing 2.5% glutaraldehyde at 4 °C for 2 h. The shells were further washed three times with a phosphate buffer and deionized water; then, they were soaked in gradually increasing concentrations of ethanol solutions (50%, 70%, 80%, 90%, and 95%). The finished shell samples were freeze-dried for 4 h and stored in a desiccator before SEM observation.

### 2.6. Statistical Analyses

All experiments were conducted at least twice, and triplicate samples were used for each test. Data were collected and analyzed by using a one-way ANOVA and Duncan’s test. The significant differences between tests were set at *p* < 0.05. All statistical analyses were performed using the SPSS program (version 12.0, St. Armonk, NY, USA).

## 3. Results

### 3.1. The Effects of Water Source, Power (watt), and Treatment Time on the Oxidation/Reduction Potential (ORP) and pH Values

**Figure 2:**
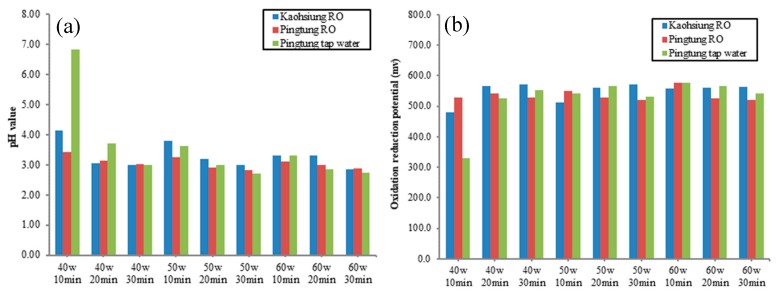
The pH values (**a**) and oxidation–reduction potential (ORP) (**b**) of the plasma-activated water (PAW) using three different water sources by various combinations of power–activation time.

After plasma activation, the water ORP values increased, but the pH values decreased due to water ionization. In addition, the degree of the ORP increase and pH decrease were correlated with the degree of water ionization [10]. Thus, the ORP and pH values were used as indicators for the PAW. During the preliminary test, the tap water in Kaohsiung showed the lowest ORP and highest pH values; precipitates were also generated during activation. The results of water hardness showed that the hardness of Kaohsiung tap water was 119.75 CaCO_3_ ppm, which was much higher than that of Pingtung tap water and that of Kaohsiung RO water, whose hardness was 15.63 and less than 2 CaCO_3_ ppm, respectively. Thus, water hardness should be a major factor for PAW production, and, subsequently, Kaohsiung tap water was excluded in this study. Higher power and a longer activation generated higher ORP and lower pH values for the PAW. Besides the 40-W, 10-min activation, no significant difference was discovered for the pH values and ORP (Figure 2). However, significant antibacterial activities were obtained with a higher power and longer activation time, as shown in Table 1. When PAW was stored at room temperature or 4 °C, its ORP value decreased significantly, but its pH values only decreased slightly, particularly at room temperature. In addition, its anti-*S. enteritidis* ability reduced rapidly during storage (Table 1). After 48 h of storage, its antibacterial activities were not significantly different from those of the sterile water control.

### 3.2. Bacterial Populations in the Suspension and on the Egg Surface after PAW Treatment

Kaohsiung RO water was used as the water source due its low hardness and availability. Furthermore, the PAW that was generated at 60 W for 20 min and used immediately after activation showed the highest reduction of *S. enteritidis* (Table 1). Thus, these combinations were used to generate the PAW used in the antibacterial test on the shell eggs. Only a 0.77 log reduction was obtained from the eggs washed with sterile water for 60 s. This indicated that the inoculated *S. enteritidis* (ATCC 13076) attached tightly to the egg’s surface. In contrast, a 4.41 log reduction were obtained from the eggs washed by the PAW over the same period of time. No significant difference was observed between 60 and 90 s of on-site treatment time. However, a significantly higher reduction (5.51 log) was obtained with 120 s of on-site treatment time (Table 2). These results indicated that the PAW should contain antibacterial substances that enable the PAW washing to achieve a 1000 times (3 log) higher reduction than the water washing. In addition, bacteria were not detected in the PAW after treatment. The physiological stages of *Salmonella* cells could affect the results of antibacterial treatments, because cells within the biofilm are more difficult to inactivate. However, the eggs studied were sent to the washing facility on the same day of laying in Taiwan. Therefore, biofilm was not a potential issue due to the short time from laying to washing.

### 3.3. Freshness Index of the Eggs

Table 3 summarizes the freshness indices under different test condition with different days of storage. The results show that there was no significant difference between the PAW treated and unwashed eggs for all indices on the same storage day. In addition, the eggs treated with PAW for 120 s maintained almost the same freshness as the eggs treated for 60 s. However, the eggs treated with commercial washing with 100 ppm chlorine showed a greater loss of freshness. On days 7 and 14, the commercially washed eggs had greater weight loss, as well as lower yolk and albumen indexes. In addition, only 2 of 3 eggs had a gravity of >1.078. On the same test day, all the PAW treated and unwashed eggs had a gravity of >1.078. 

### 3.4. Scanning Electronic Microscope (SEM) Observation

**Figure 3:**
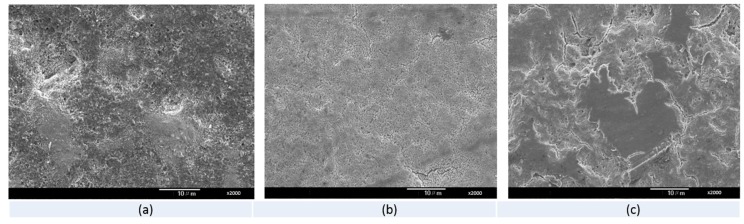
Appearance of the egg surface under SEM after different treatments. (**a**) Unwashed; (**b**) PAW treated; (**c**) Commercially washed.

**Figure 4:**
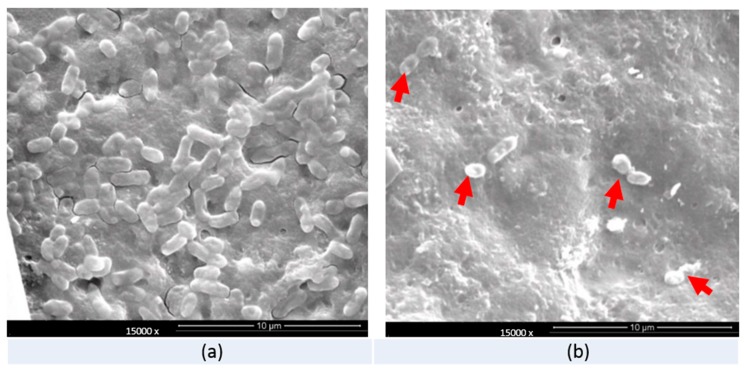
The SEM observation of the *S. enteritidis* (ATCC 13076) on the surface of the unwashed or PAW treated eggs (60 w, 60 s). (**a**) Unwashed control; (**b**) PAW treatment.

Figure 3 shows the SEM images of the surface cuticles of the eggs before and after treatment by different solutions. The results show that the surfaces remained relatively intact on the unwashed and the PAW treated eggs. However, severe damage was observed on the surface of the commercially washed eggs on which most of the top layers of cuticles disappeared. In contrast, only the pits and cracks were observed on the surface of the unwashed and PAW treated eggs. Figure 4 shows that, for the observation of *S. enteritidis* on the eggs’ surfaces, much less bacteria were observed under SEM on the surfaces of the PAW treated eggs compared to the control case. In addition, the bacterial cells on the surface of the PAW treated eggs were clearly deformed or broken.

## 4. Discussions

This study is the first report to use air PAW to inactivate *S. enteritidis* on shell eggs. Among the reported studies applying PAW, an inactivation of *Salmonella* spp. was only tested on beef, to which plasma-activated lactic acid was applied, and a 1.24–3.52 log CFU/g reduction of *S. enteritidis* was obtained [16]. Our study showed more than a 4 log reduction of *S. enteritidis* after 60 s of on-site PAW treatment. Although plasma activated lactic acid theoretically possesses higher antibacterial activity than PAW, the higher reduction we obtained could be due to the lesser quantity of organic matter on the egg’s surface than of the beef’s. Xiang et al. (2019) reported when beef extract was added into a suspension of *E. coli* O157:H7 and *Staphylococcus aureus*, the antibacterial activity of PAW decreased dramatically [19]. Therefore, Xiang et al. (2019) concluded that organic matter could hinder the antibacterial ability of PAW [19]. The reductions of pathogens in the other examples using PAW to inactivate pathogens on food surfaces also had results less than 4 log. Besides the less organic matter and relatively smooth surface of the eggs, different bacteria were tested. *Staphylococcus aureus* and natural microorganisms were tested in studies of strawberry [15] and mushroom [10], respectively. Combining with mild heat at 60 °C, 3.4 and 3.7 log reductions of *S. aureus* and *Listeria monocytogenes* were obtained on the cabbages used in Korean kimchi [20]. The ongoing studies in our laboratory also found that *Salmonella* spp. seemed to be more sensitive to PAW than other bacteria, such as *E. coli* and *L. monocytogenes*.

Our results showed less damage on the eggs washed by PAW than on the commercially washed eggs. The egg cuticle, which comprises several layers, is closely associated with egg freshness (Bagheri et al., (2019)) [18]. Thus, less damage to the cuticle usually indicates a lower loss of egg quality during storage. Georgescu et al. (2017) and Dasan et al. (2018) reported that the non-thermal atmospheric plasma treatment caused no or very slight damage to the cuticles on the eggs [21,22]. However, we did not compare the plasma-treated eggs with the commercially treated ones in both groups. This study clearly showed that PAW caused less damage than commercial treatment, and the quality indices of the PAW-treated eggs were better than those of the commercially treated ones. SEM observation also revealed that the inoculated bacteria were deformed or showed severe surface damage after PAW treatment. Severe morphological changes and significantly higher permeability were observed for *Pseudomonas deceprionenesis* treated with PAW for 10 min [19]. Hence, membrane damage could be the main cause for the death of bacteria treated with PAW. Acidic pH, higher ORP, H_2_O_2_, nitrate, and nitrite anions generated by plasma in the water were indicated to be the mechanisms behind the antibacterial activities of PAW. Thirumdas et al. (2018) [19] also suggested that the O_3_, H_2_O_2_, nitrate, and nitrite generated in PAW were the main causes for the antibacterial ability. During this study, O_3_, H_2_O_2_, nitrate, and nitrite were detected by qualitative tests. Comprehensively quantitative tests are currently being conducted with a larger quantity (1 L) in our laboratory. Therefore, more detailed information will be obtained to better understand the antibacterial mechanism and antibacterial efficacy of PAW in large quantities.

In this study, the longer activation and treatment time of PAW on eggs resulted in a higher reduction of microorganisms, but storage decreased these antibacterial activities. These results are similar to those of previous studies [12]. However, our system was more simple and had a lower operating cost than that of other PAW systems, which used silver electrodes [23], Ar gas [24], Ar and oxygen gas [15], or four electrodes [13]. Our study is also the first report to indicate that the hardness of water could be a critical criterion for choosing the water source to produce PAW. In the study, the low hardness water obtained by ion exchanging showed inferior results. The possible reason for this result could be the high amount of sodium ions in the water, because calcium and magnesium ions were exchanged by sodium ions, which could hinder the ionization of the PAW process.

The commercial process for egg washing usually includes washing using chlorinated water, drying, UV sterilization, checking cracks, and packaging. The washing step is the most important step to remove debris and microorganisms on the egg’s surface. Based on our study, PAW treatment could be an effective process to effectively inactivate *S. enteritidis* and preserve the freshness of eggs. Although the eggs treated with PAW for 120 s had a greater log reduction (by one) of *Salmonella* than the one treated for 60 s, the populations of contaminated *Salmonella* on the egg’s surface were less than 5 log in most natural situations. In addition, washing for 60 s is much more practical in the egg industry than washing for 120 s. Compared with the current commercial practice, in which most of the electrical energy is used to increase the water temperature, the electricity cost of the PAW system in this study is about the same. The results of this study demonstrate that a PAW generation system could be integrated into pre-existing commercial washing systems as an alternative process to chlorine washing. 

## 5. Conclusions

In this study, we applied a patented air plasma jet to generate plasma-activated water under various water sources, treatment times, and input powers. The antibacterial efficacy of these PAWs on *S. enteritidis* was tested. The optimal test condition used to generate PAWs was further applied to inactivate *S. enteritidis* inoculated on egg surfaces. The results showed that low hardness water, such as RO water, was the optimal water source. In addition, the optimal test condition was found to be 60 W of power and 20 min of activation. Increasing the power and activation time was able to decrease the pH value, increase ORP, and thus lower the antibacterial activity of PAW. The combination of RO water, 60 W, and 20 min was used to inactivate *S. enteritidis* on the egg’s surface. More than a 4 log reduction was obtained after 60 s of treatment, and the freshness indices of the PAW-treated eggs were better than those of the commercially treated ones. Observation under SEM revealed less damage of the cuticles on PAW-treated eggs than on the commercially treated ones. In addition, *S. enteritidis* cells were deformed or broken after PAW treatment. The results of this study indicate that air PAW treatment could be a better option to clean and disinfect shell eggs than current commercial processes. 

## Figures and Tables

**Table 1:** Population (log CFU/mL) of *S. enteritidis* (ATCC 13076), pH, and ORP (mv) of Kaohsiung RO water after different plasma treatments and storage days. CFU: colony-forming units, RO: reverse osmotic.

	Storage Days (Day 0)			Storage Days (Day 1)		
Treatment	Bacterial Population	ORP	pH	Bacterial Population	ORP	pH
Sterile water	5.98 ± 0.35 ^dA^	348.7 ± 9.1 ^aA^	6.50 ± 0.08 ^aA^	5.77 ± 0.63 ^dA^	396.3 ± 8.93 ^aA^	6.49 ± 0.15 ^aA^
40 w 30 min	5.27 ± 0.23 ^cA^	517.1 ± 12.3 ^bA^	3.05 ± 0.16 ^bA^	5.41 ± 0.32 ^cA^	449.6 ± 10.4 ^bB^	2.80 ± 0.23 ^bA^
50 w 20 min	4.90 ± 0.21 ^bA^	556.7 ± 11.3 ^bA^	3.19 ± 0.11 ^bA^	4.82 ± 0.25 ^bA^	504.4 ± 12.5 ^bB^	2.92 ± 0.16 ^bA^
60 w 10 min	3.88 ± 0.13 ^bA^	569.6 ± 8.3 ^bA^	3.35 ± 0.20 ^bA^	4.62 ± 0.14 ^bB^	516.8 ± 12.4 ^bB^	3.07 ± 0.18 ^bA^
60 w 20 min	2.18 ± 0.10 ^aA^	567.5 ± 10.3 ^bA^	3.13 ± 0.14 ^bA^	3.63 ± 0.33 ^aB^	532.5 ± 11.5 ^bB^	2.89 ± 0.15 ^bA^

Data with different lower-case letters in the same column are significantly different (*p* < 0.05). The data of different days with upper case letters are significantly different (*p* < 0.05). The testing PAW was stored at 4 °C.

**Table 2:** Populations (log CFU/egg) of *S. enteritidis* (ATCC 13076) on the surfaces of eggs with different PAW treatment times.

Treatments	Bacterial Populations	Reduction
Unwashed	7.68 ± 0.13 ^e^	
Sterile water 60 s	6.91 ± 0.18 ^d^	0.77
PAW 30 s	5.30 ± 0.16 ^c^	2.38
PAW 60 s	3.28 ± 0.31 ^b^	4.40
PAW 90 s	3.69 ± 0.12 ^b^	3.99
PAW 120 s	2.17 ± 0.17 ^a^	5.51

The RO water in Kaohsiung was used, and the PAW was activated at 60 W for 20 min before testing. Data are shown as the average ± standard deviation. Data with different letters in the same column are significantly different (*p* < 0.05).

**Table 3:** Freshness indices for the eggs with different treatments on day 0, 7, and 14 after treatment.

Treatments	Storage Day	Weight Loss (%)	Specific Gravity ^A^	Yolk Index	Albumen Index
PAW—60 s	0	0	3	0.40 ± 0.02 ^a^	0.10 ± 0.02 ^a^
	7	0.28 ± 0.00 ^a^	3	0.33 ± 0.01 ^b^	0.06 ± 0.01 ^b^
	14	0.69 ± 0.04 ^b^	3	0.22 ± 0.02 ^c^	0.06 ± 0.02 ^b^
PAW—120 s	0	0	3	0.41 ± 0.03 ^a^	0.10 ± 0.02 ^a^
	7	0.29 ± 0.02 ^a^	3	0.32 ± 0.01 ^b^	0.07 ± 0.02 ^b^
	14	0.66 ± 0.06 ^b^	3	0.21 ± 0.03 ^c^	0.06 ± 0.01 ^b^
Commercially washed	0	0	3	0.38 ± 0.02 ^a^	0.08 ± 0.03 ^a^
	7	0.68 ± 0.06 ^b^	2	0.21 ± 0.01 ^c^	0.06 ± 0.00 ^b^
	14	1.06 ± 0.08 ^c^	2	0.18 ± 0.03 ^d^	0.04 ± 0.00 ^c^
unwashed	0	0	3	0.39 ± 0.09 ^a^	0.09 ± 0.04 ^a^
	7	0.31 ± 0.03 ^a^	3	0.32 ± 0.01 ^b^	0.06 ± 0.00 ^b^
	14	0.72 ± 0.04 ^b^	3	0.25 ± 0.00 ^c^	0.05 ± 0.04 ^b^

The RO water in Kaohsiung was used, and the PAW was activated at 60 W for 20 min before testing. Data are shown as the average ± standard derivation of the triplicate samples of two tests. ^A^ The number in this column indicates the number of eggs whose gravity was >1.078. Data with different letters in the same column are significantly different (*p* < 0.05).
